# Mg^2+^ Transporters in Digestive Cancers

**DOI:** 10.3390/nu13010210

**Published:** 2021-01-13

**Authors:** Julie Auwercx, Pierre Rybarczyk, Philippe Kischel, Isabelle Dhennin-Duthille, Denis Chatelain, Henri Sevestre, Isabelle Van Seuningen, Halima Ouadid-Ahidouch, Nicolas Jonckheere, Mathieu Gautier

**Affiliations:** 1 Université de Picardie Jules Verne, UFR des Sciences, UR-UPJV 4667, F-80000 Amiens, France; julie.auwercx@etud.u-picardie.fr (J.A.); Rybarczyk.Pierre@chu-amiens.fr (P.R.); philippe.kischel@u-picardie.fr (P.K.); isabelle.dhennin@u-picardie.fr (I.D.-D.); sevestre.henri@chu-amiens.fr (H.S.); halima.ahidouch-ouadid@u-picardie.fr (H.O.-A.); 2 Service d’Anatomie et Cytologie Pathologique, CHU Amiens-Picardie, F-80000 Amiens, France; chatelain.denis@chu-amiens.fr; 3 Univ. Lille, CNRS, Inserm, CHU Lille, UMR9020-U1277—CANTHER—Cancer Heterogeneity Plasticity and Resistance to Therapies, F-59000 Lille, France; isabelle.vanseuningen@inserm.fr (I.V.S.); nicolas.jonckheere@inserm.fr (N.J.)

**Keywords:** magnesium transporters, digestive cancers, TCGA, overall survival

## Abstract

Despite magnesium (Mg^2+^) representing the second most abundant cation in the cell, its role in cellular physiology and pathology is far from being elucidated. Mg^2+^ homeostasis is regulated by Mg^2+^ transporters including Mitochondrial RNA Splicing Protein 2 (MRS2), Transient Receptor Potential Cation Channel Subfamily M, Member 6/7 (TRPM6/7), Magnesium Transporter 1 (MAGT1), Solute Carrier Family 41 Member 1 (SCL41A1), and Cyclin and CBS Domain Divalent Metal Cation Transport Mediator (CNNM) proteins. Recent data show that Mg^2+^ transporters may regulate several cancer cell hallmarks. In this review, we describe the expression of Mg^2+^ transporters in digestive cancers, the most common and deadliest malignancies worldwide. Moreover, Mg^2+^ transporters’ expression, correlation and impact on patient overall and disease-free survival is analyzed using Genotype Tissue Expression (GTEx) and The Cancer Genome Atlas (TCGA) datasets. Finally, we discuss the role of these Mg^2+^ transporters in the regulation of cancer cell fates and oncogenic signaling pathways.

## 1. Introduction

According to the International Agency for Research, digestive cancers are the most common and deadliest malignancies worldwide [1]. In this review, we choose to focus on the main digestive cancers namely esophageal adenocarcinoma, gastric cancer, pancreatic ductal adenocarcinoma and colorectal cancer.

Esophageal cancer (ESAC) is ranked in the seventh position in terms of incidence and in the sixth in terms of mortality [1]. ESAC is the most common type of esophageal cancer in industrialized countries [2]. It is among the most lethal digestive malignancies with only 16% of patients surviving 5 years after diagnosis and a median survival that is less than 1 year [3]. The main risk factor for ESAC is the gastroesophageal reflux disease, that leads to inflammation of esophageal and remodeling of tissue into a metaplastic, specialized intestinal epithelium named Barrett’s esophagus. Tobacco smoking and obesity have been also identified as others strong risk factors for ESAC [3].

Gastric cancer (GC) is the fifth most common cancer worldwide and the third deadliest [1]. The 5-year survival rate is dependent of the stage of disease at the diagnosis. GCs detected at early stage have a 5-year survival rate around 80% [4]. There is strong evidence that *Helicobacter pylori* infection is a risk factor for GC development, therefore *Helicobacter pylori* has been classified as a class I carcinogen by International Agency for Research on Cancer [5]. As for many cancers, dietary factors play also a role in stomach carcinogenesis. Fruits, vegetables, and vitamins intake seem to have a protective role, while alcohol, coffee, meat and high salt consumption seem to increase the risk of developing GC.

Pancreatic ductal adenocarcinoma (PDAC) is the 7th leading cause of global cancer deaths in industrialized countries and the 3rd in USA, while it is ranked in the 11th position in term of incidence [6]. Unlike most cancers, the PDAC incidence is in constant progression and it is estimated that it will become the second deadliest cancer in 2030 [7]. The poor prognosis associated with PDAC is because this malignancy is mainly diagnosed too late in an advanced and metastatic stage. To date, carbohydrate antigen 19-9 (CA 19-9) is the only diagnostic marker for PDAC approved by the U.S. Food and Drug Administration (FDA). However, other cancers and benign diseases can cause CA 19-9 overexpression which can explain the poor specificity. Thus, there is an urgent need for specific biomarkers for PDAC [8]. To date, cigarette smoking and family history are the main risk factors but dietary style and obesity have been also considered [6].

Colorectal cancer (CRC) is at the third rank in term of incidence and at the second rank in term of mortality [1]. The incidence of CRC is country-dependent and the main factor risks for CRC are family hereditary, red and processed meat consumption, alcohol drinking, obesity, and inflammatory bowel disease [9]. Surprisingly, while the overall incidence and mortality are decreasing, the incidence of early-onset CRC, generally diagnosed before 50 years old, is increasing worldwide. The increase in early-onset CRC incidence associated with a higher mortality rate for young adults may be associated with Western lifestyle, including diet [9]. Consequently, there is an important role of nutrition in cause and prevention of CRC [10].

Nutrients are transported through the gastrointestinal tract and nutrient deficiency could be associated with digestive cancer initiation and/or promotion. Among these nutrients, low magnesium intake is observed in a large part of the population, especially in industrialized countries. The aim of this review is to present the current knowledge on magnesium levels and digestive cancer development. Firstly, we will focus on magnesium transporter expression in digestive cancers by analyzing the Cancer Genome Atlas (TCGA). In the last part, the role of these magnesium transporters in cancer cell fate and their potential importance as new biomarkers in digestive cancers will be discussed.

## 2. Magnesium

Magnesium (Mg^2+^) is one of the most important ions in health and is the second most abundant cation in the cell with a concentration estimated between 10 and 30 mM. Due to the binding to different partners like ATP, ribosomes, or nucleotides, the free intracellular Mg^2+^ levels lower to 0.5 to 1.2 mM [11]. Mg^2+^ is essential in almost all cellular processes, acting as a cofactor and activator for various enzymes [11]. For example, Mg^2+^ is essential in DNA stabilization, DNA repair mechanisms, or even protein synthesis [12,13,14,15]. New interactions are still being discovered, expanding the importance of this cation [16].

**Figure 1:**
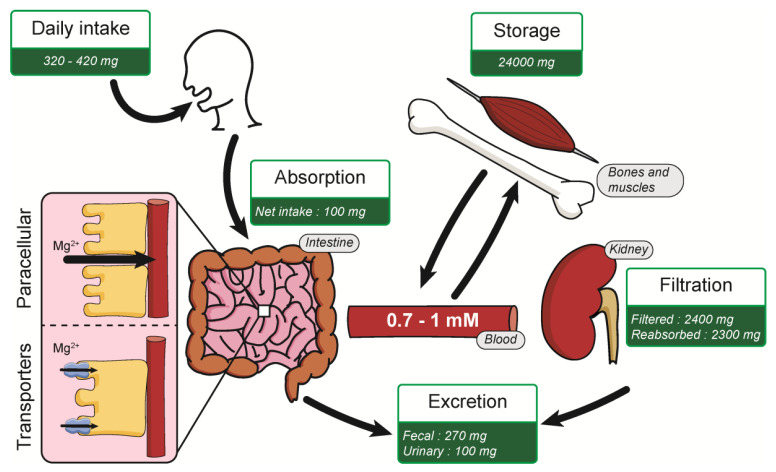
Summary of Mg^2+^ homeostasis.

Normal Mg^2+^ in blood serum levels for healthy people is about 0.7–1 mM, corresponding to an average daily intake (ADI) of 320–420 mg/day [17,18]. This Mg^2+^ intake is absorbed mostly in the small intestine by two mechanisms: paracellular transport and via the expression of membrane transporters (Figure 1). Paracellular transport is predominant, mainly because of low expression of claudins in the small intestine [19,20]. Numerous Mg^2+^ transporters are also present in the plasma membrane of intestine cells for Mg^2+^ absorption. An average of 100 mg is absorbed in the intestine, depending on the daily Mg^2+^ intake [11]. Kidneys filters around 2400 mg of Mg^2+^ per day in the glomeruli, where most of the Mg^2+^ (2300 mg) is reabsorbed in the thick ascending limb of Henle’s loop. Mg^2+^ is mainly stored in bones but also in muscles and soft tissues. [11,21]. This organization allows the Mg^2+^ homeostasis balance, maintaining a constant 0.7–1 mM Mg^2+^ serum level in normal conditions.

Unfortunately, our alimentation contains nowadays less Mg^2+^ because of the development of the food industry and changes in soils due to intensive farming [22,23]. Along with the modifications of our eating habits and the prevalence of processed foods, it is shown that a large number of adults do not reach the recommended Mg^2+^ average daily intake [24]. Hypomagnesemia is characterized by Mg^2+^ serum levels <0.7 mM, but it is often underestimated because the serum levels are not representative of the whole Mg^2+^ availability [25]. Hypomagnesemia is associated with several health issues such as epilepsy, cystic fibrosis, atherosclerosis, and type 2 diabetes [26,27,28,29].

Several studies suggest that calcium (Ca^2+^) and Mg^2+^ can compete during intestinal absorption, leading to the consideration also of the Ca^2+^/Mg^2+^ ratio for assessing Ca^2+^ and Mg^2+^ intakes [30].

**Figure 2:**
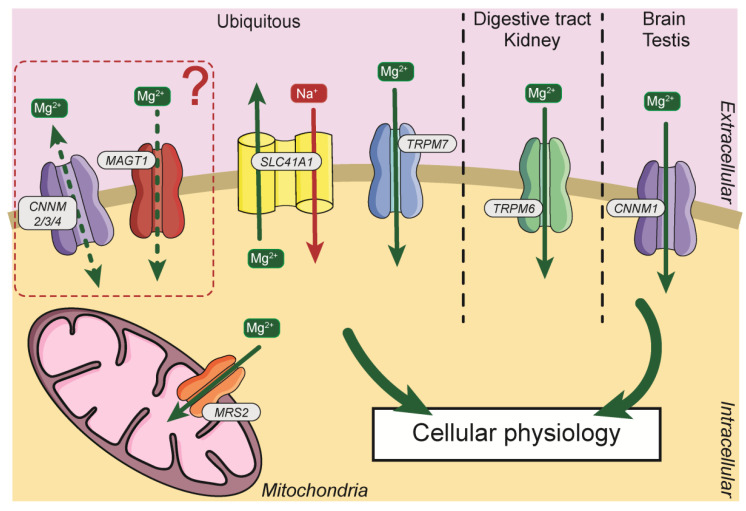
General distribution and localization of Mg^2+^ transporters in cells. Mg^2+^, magnesium; Na^+^, sodium; CNNM2/3/4, Cyclin and CBS Domain Divalent Metal Cation Transport Mediator2/3/4; MAGT1, Magnesium Transporter 1; SLC41A1, Solute Carrier Family 41, Member 1; TRPM7, Transient Receptor Potential Cation Channel Subfamily M Member 7; TRPM6, Transient Receptor Potential Cation Channel Subfamily M Member 6; CNNM1, Cyclin and CBS Domain Divalent Metal Cation Transport Mediator1; MRS2, Mitochondrial RNA Splicing Protein 2.

Due to its importance, Mg^2+^, requires a specific transport system. The first magnesium transporters were identified in prokaryotes, with the identification of the proteins magnesium/cobalt transporter (CorA), magnesium-transporting ATPase (MgtA/B/E) [31]. Subsequently, Mg^2+^ transporters were identified and cloned in other models (Figure 2). In Mammals, several transporters have been identified and will be described in this manuscript.

### 2.1. MRS2

The first Mg^2+^ transporter characterized in Metazoa is the Mitochondrial RNA splicing protein 2 (MRS2/MRS2p). It was discovered as a CorA homolog, localized in the mitochondrial inner membrane, and involved in Mg^2+^ mitochondrial uptake. A ubiquitous mRNA expression of MRS2 was found in rat tissues [32]. With the use of a MagFura-2 fluorescent probe in yeast, MRS2 overexpression was shown to enhance mitochondrial Mg^2+^ influx [33]. On the other hand, the mitochondrial Mg^2+^ influx was abolished upon MRS2 gene deletion. The MRS2 protein is therefore described as an essential magnesium transporter in the mitochondria.

### 2.2. TRPM7 and TRPM6

The transient receptor potential cation channel subfamily M member 7 (TRPM7) was discovered and cloned by two teams under different names, Long TRP Channel 7 (LTRPC7) and TRP-Phospholipase C Interacting Kinase (TRP-PLIK). TRPM7 is first known as the long transient receptor potential channel 7 (LTRPC7), a member of LTRPCs by its similarity with the first 1200 amino-acids [34]. Its carboxy-terminal tail is pretty unique as it contains a kinase domain, with significant homology to the protein-kinase family of Myosin Heavy Chain Kinase/Eukaryotic Elongation Factor 2 Kinase (MHCK/eEF-2) [35]. In the DT-40 lymphoma cell line, TRPM7 has a role in viability and proliferation [34]. Using the patch-clamp technique in the HEK-293 cell line, it was shown that LTRPC7 was permeable to Ca^2+^ and Mg^2+^ and was inhibited by cytosolic free Mg^2+^ and magnesium bound to ATP (MgATP) [34]. In the same year, the TRP-PLIK was described, with similarity with the LTRPC family [36]. TRP-PLIK, with its kinase domain, is suspected to have autophosphorylation properties. Using patch-clamp on CHO-K1 cells, it was shown that TRP-PLIK is permeable to Ca^2+^ and monovalent cations like sodium (Na^+^) or potassium (K^+^). Other electrophysiological studies on HEK-293 cells have shown that the TRPM7 channel is also permeable to other cations like zinc, nickel, baryum, cobalt, strontium, and cadmium [37]. TRPM7 expression was found to be ubiquitous in mouse and human tissues [36,38]. TRPM7 is now proposed as an essential actor in magnesium homeostasis, embryonic development, and mineral absorption [39,40,41].

The Transient Receptor Potential Cation Channel Subfamily M, Member 6 (TRPM6) is the second TRPM channel involved in Mg^2+^ transport. The mutated gene is associated with “hypomagnesemia with secondary hypocalcemia” (HSH) [42,43]. TRPM6 shares strong homology with TRPM7, and also has an alpha-kinase domain at its C-terminus [35]. Strong TRPM6 mRNA expression was found in the intestine and the distal convoluted tube (DCT) in mouse kidney tissues, and this expression was confirmed in human tissues [44]. The protein was detected at the apical membrane of DCT in mouse kidney and the brush-border membrane of the small intestine. Using a patch-clamp, it was shown that TRPM6 is responsible for Mg^2+^ currents [44] and TRPM6 is now considered as an essential actor in Mg^2+^ (re)absorption in the kidney.

### 2.3. SLC41A1

The human transporter solute carrier family 41, member 1 (SLC41A1) was also identified by homology with a prokaryote Mg^2+^ transporter, the Magnesium Transporter E (MgtE). Both transporters share similarities on two transmembrane domains [45]. SLC41A1 expression is ubiquitous, with highest expression in heart and testis tissues. By using a patch-clamp in Xenopus oocytes, SLC41A1 was identified as a voltage-dependent Mg^2+^ transporter and is also permeable to other divalent cations such as cobalt, copper, and zinc [46]. Interestingly, upregulation of SLC41A1 transcripts has been observed following hypomagnesemia in mouse kidney tissues [46]. Based on Mg^2+^ imaging on HEK293 cells, SLC41A1 is now identified as a Na^+^/Mg^2+^ exchanger that allows Mg^2+^ efflux [47].

### 2.4. MAGT1

The Magnesium Transporter 1 (MAGT1) was identified as a gene upregulated in conditions of Mg^2+^ deficiency [48]. This transporter is voltage-dependent and involved in Mg^2+^ uptake when expressed in Xenopus oocytes. Unlike the other Mg^2+^ transporters, MAGT1 is able to achieve a specific transport. Its expression is ubiquitous in all human tissues. It is also essential for the development of zebrafish, underlying a role in vertebrate embryonic development [49]. Mg^2+^ was suspected as a second messenger in the X-linked human immunodeficiency with Mg^2+^ defect and Epstein–Barr virus infection and neoplasia (XMEN): it appeared that mutations in the MAGT1 gene were actually involved [50]. In disorders like XMEN and congenital disorders of glycosylation (CDG), these mutations caused N-glycosylation defects [51]. MAGT1 has been recently identified as a member of glycoside complexes, regulated by Mg^2+^ [52].

### 2.5. CNNM Family

The Cyclin and CBS Domain Divalent Metal Cation Transport Mediator (CNNM) family was first known as the Ancient Conserved Domain Protein (ACDP) family by the conserved domain structures among different species like yeasts, bacteria, and others like *Drosophilia Melanogaster* [53]. The ACDP family has four members, ACDP1/2/3/4 (corresponding to CNNM1/2/3/4), that share a minimum of 62.8% nucleotide similarity. It has been shown by Northern-blotting that ACDP2/3/4 are found in all human tissues, while ACDP1 is found mostly in brain and testis tissues. Other works found out that mouse and human ACDP were similar in structure and tissue distribution [54].

The two most studied members of the ACDP/CNNM family are ACDP2/CNNM2 and ACDP4/CNNM4. By studying CNNM2 in Xenopus oocytes, it has been defined that this protein was a cation transporter for magnesium, cobalt, manganese, strontium, baryum, and copper [55]. CNNM2 mRNA was also regulated by Mg^2+^ deficiency in distal convoluted tubule (MDCT) epithelial cells. However, the role of CNNM2 as a Mg^2+^ transporter is still debated because of its Mg^2+^ sensitivity and transport capacity in HEK293 cells [56].

Th role of CNNM4 was firstly studied in rat spinal cord dorsal horn neurons, where it interacts with the Cytochrome C Oxidase Copper Chaperone 11 (COX11) [57]. Since its overexpression in HEK293 cells causes Cu^2+^, Mn^2+^, and Co^2+^ toxicity, CNNM4 was suggested as a divalent cation transporter. Other studies localized CNNM4 on the basolateral side of intestinal epithelial cells, where it extrudes Mg^2+^. Mice lacking CNNM4 also show hypomagnesemia and Jalili syndrome, characterized by cone–rod dystrophy and amelogenesis defect [58]. However, the role of CNNM4 as a Mg^2+^ transporter or a Na^+^/Mg^2+^ is still discussed [58,59].

## 3. Mg^2+^ Intake and Digestive Cancers

There is much evidence suggesting an association between Mg^2+^ intake and digestive cancer risk and/or development. For example, high Mg^2+^ intake and particularly low Ca^2+^/Mg^2+^ ratio protects against reflux esophagitis and Barret’s esophagus, two precursors of ESAC. However, no significant associations were observed between Mg^2+^ intake and ESAC incidence [60]. However, the association is less evident for GC because there is only a suggestive trend for a preventive effect of high Mg^2+^ intake in non-cardia GC depending of gender and dietary source of Mg^2+^ [61].

In PDAC, a first study from 2012 in a large cohort (142,203 men and 334,999 women) recruited between 1992 and 2000 shows no association between Mg^2+^ intake and cancer risk [62]. Another study has investigated the association between nutrients intake from fruit and vegetable and PDAC risk [63]. The results show an inverse association between PDAC risk and nutrient intake, including Mg^2+^, in a dose-dependent manner. Importantly, Dibaba et al. have shown in a large cohort, followed from 2000 to 2008, that every 100 mg per day decrement in Mg^2+^ intake was associated with a 24% increase in PDAC incidence [64]. Moreover, analysis of metallomics in PDAC reveals a lower concentration of Mg^2+^ in urine of patients with PDAC [65].

Mg^2+^ intake was associated with a lower risk for CRC, particularly in people with low Ca^2+^/Mg^2+^ intake ratio [66]. Importantly, Dai et al. also show that the *Thr1482Ile* polymorphism in the *TRPM7* gene increases the risk for adenomatous and hyperplastic polyps [66]. It was also shown that Mg^2+^ intake around 400 mg per day has a protective effect for CRC incidence in postmenopausal women [67]. A meta-analysis from 29 studies published on PubMed, Web of Science and the Chinese National Knowledge Infrastructure confirms that the high intake of Mg^2+^ is inversely associated with the risk of CRC [68]. Assessment of Mg^2+^ concentration in serum showed an inverse association with CRC risk in female but not in male. Moreover, no significant association was detected between dietary Mg^2+^ and CRC risk in this study [69]. Finally, Wesselink et al. suggested that an interaction between normal 25-hydroxyvitamin D_3_ concentration and high Mg^2+^ intake is essential for reducing the risk of mortality by CRC [70].

To summarize, these epidemiologic studies suggested that high Mg^2+^ intake by diet and/or supplemental compounds is inversely associated with CRC, PDAC and possibly ESAC risk, but not with GC risk.

## 4. Expression of Mg^2+^ Transporters in Digestive Cancers

Ion channels are essential for physiological function of the digestive system. Although some of these (e.g., chloride, potassium, calcium, sodium and zinc) are dysregulated in cancer [71,72], the expression of Mg^2+^ transporters in digestive cancers is less extensively studied.

### 4.1. Analysis of the Literature 

In ESAC, immunohistochemistry (IHC) analyses have shown that TRPM7 protein was expressed in the cytoplasm of carcinoma cells while not detected in the non-cancerous esophageal epithelia. High TRPM7 staining was associated with better 5-year survival in patients with ESAC [73] (Table 1).

In PDAC patients, TRPM7 protein was overexpressed in cancerous tissues when compared to normal adjacent tissues [74,75,76,77]. TRPM7 expression correlates with tumor size, grade, and a high expression of this protein associates with a poor prognosis in patients [75,76]. On the other hand, SLC41A1 protein and mRNA were downregulated in PDAC patients compared to normal adjacent tissues, using quantitative real time-PCR (qRT-PCR), IHC, and in silico studies [78]. SLC41A1 expression is inversely correlated with tumor grade and was positively associated with a better outcome for patients [78].

In CRC, the expression of Mg^2+^ transporters has been investigated in numerous studies. Evaluation of TRPM6 expression at the mRNA level (by qRT-PCR and in TCGA datasets), it has been found that TRPM6 was downregulated in cancerous colorectal tissues and that a high TRPM6 expression correlates with better survival in patients [79,80]. However, overexpression of TRPM6 protein was observed by IHC on several colorectal cancerous tissues when compared to matched normal tissues [81]. TRPM7 was also found upregulated in CRC using in silico datasets but also qRT-PCR, immunofluorescence, and IHC on tissues. TRPM7 expression was also associated with tumor infiltration, tumor grade, and the presence of distant metastasis [81,82]. In qRT-PCR-based and in silico studies, the Mg^2+^ transporter MAGT1 was found to be overexpressed in colorectal cancerous tissues [83]. High MAGT1 expression also correlates with chemotherapeutic resistance, metastatic status, and tumor stage [83]. CNNM4 protein was also found downregulated in an IHC analysis of cancerous colorectal tissues, and inversely correlates with tumor malignancy [84].

### 4.2. Analysis of the Human Protein Atlas

The analysis of the Protein Atlas program provides some interesting data on IHC staining of Mg^2+^ transporters on paraffin-embedded tumoral tissue sections (Table 2). Most transporters are expressed in PDAC, CRC and GC. However, TRPM7 is only found in GC, while TRPM6 and CNNM2 are not detected. A homogenous moderate to strong staining is found for SCL41A1 and MRS2, whereas MagT1 staining appears more heterogeneous. These results need to be confirmed in larger cohorts, because the number of studied cases varies currently from 8 to 12. Although staining intensity of cancer cells is analyzed, the difference between normal and peritumoral tissues is not systematically considered. These data provide preliminary results on Mg^2+^ transporters in digestive cancer tissues, but they still need to be confirmed in larger cohorts and by a comparative study with non-cancerous or healthy tissues.

### 4.3. Transcriptome Analysis in Datasets

We analyzed Mg^2+^ transporters’ expression in digestive cancers, correlation and their impact on patient overall and disease-free survival using Genotype Tissue Expression (GTEx) and The Cancer Genome Atlas (TCGA) datasets using GEPIA2 and RStudio tools, as previously performed [86].

#### 4.3.1. Mg^2+^ Transporters Expression in Digestive Cancers

We investigated Mg^2+^ transporters expression using available datasets. Whisker boxplots of Mg^2+^ transporters mRNA (TRPM6, TRPM7, MAGT1, SLC41A1, MRS2, CNNM1, CNNM2, CNNM3, CNNM4) were generated using GEPIA2, that compiles GTEx and TCGA datasets of normal and tumoral samples from the different digestive organs of interest (Supplementary Figure S1).

**Figure 3:**
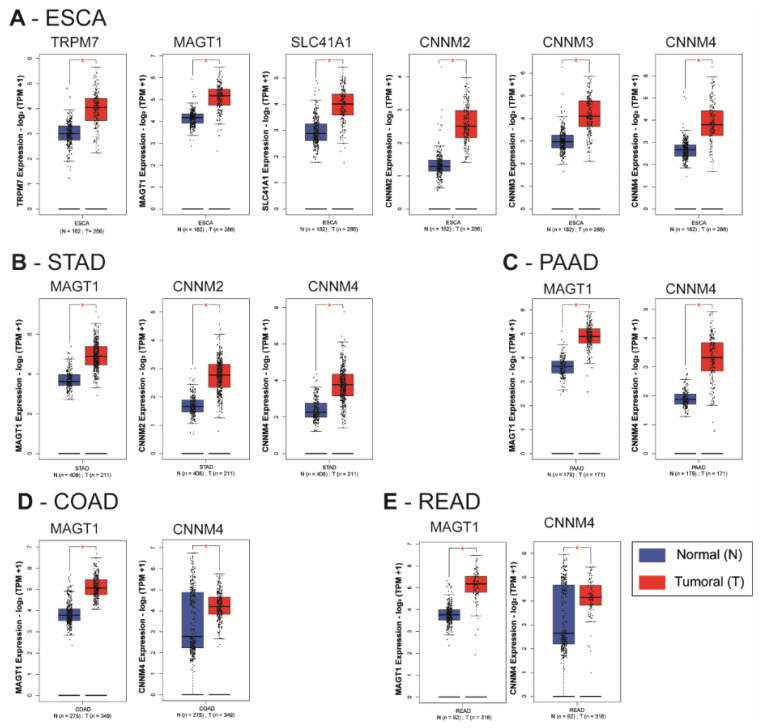
Relative mRNA expression of magnesium transporters in digestive cancers and normal tissues. Whiskers boxplots for Mg^2+^ transporters mRNA (TRPM6, TRPM7, MAGT1, SLC41A1, MRS2, CNNM1, CNNM2, CNNM3, CNNM4) were generated using GEPIA2 from The Cancer Genome Atlas (TCGA) and Genotype-Tissue Expression (GTEx) samples. TCGA datasets were (**A**) Esophageal carcinoma (ESCA), (**B**) Stomach Adenocarcinoma (STAD), (**C**) Pancreatic Adenocarcinoma (PAAD), (**D**) Colon Adenocarcinoma (COAD) and (**E**). Rectum Adenocarcinoma (READ). TRPM7, Transient Receptor Potential Cation Channel Subfamily M, Member 7; MAGT1, Magnesium Transporter 1; SLC41A1, Solute Carrier Family 41, Member 1; MRS2, Mitochondrial RNA Splicing Protein 2; CNNM4, Cyclin and CBS Domain Divalent Metal Cation Transport Mediator 4; *n* = number of samples; N, normal; T, tumoral. Relative mRNA levels are expressed as log2 transcripts per million bases (TPM). Only significative results (* *p* < *0.01*) are presented. The whole dataset analysis is provided as supplementary data (Supplementary Figure S1).

We observed a statistically significant overexpression of the transporters TRPM7, MAGT1, SLC41A1, CNNM2, CNNM3, and CNNM4 in the esophageal carcinoma (ESCA) samples when compared to corresponding normal tissues (*p* < 0.01) (Figure 3A). MAGT1, CNNM2 and CNNM4 mRNA were increased in tumoral tissues of stomach adenocarcinoma (STAD) when compared to normal stomach tissues (*p* < 0.01) (Figure 3B). In pancreatic adenocarcinoma (PAAD), colon adenocarcinoma (COAD) and rectum adenocarcinoma (READ), MAGT1 and CNNM4 mRNA relative levels were increased when compared to their normal corresponding samples (*p* < 0.01) (Figure 3C–E). A limitation of this type of transcriptome analysis is the homogeneity variances and related robustness of the analysis. This is why it will be important to perform additional studies on independent datasets for each digestive cancer as well as analyzing formalin-fixed paraffin-embedded (FFPE) samples by IHC. Moreover, characterization of gene of interest expression in pathological stages or other clinical features might reinforce the involvement of each magnesium transporter during carcinogenesis progression.

#### 4.3.2. Mg^2+^ Transporters and Patient Survival

**Figure 4:**
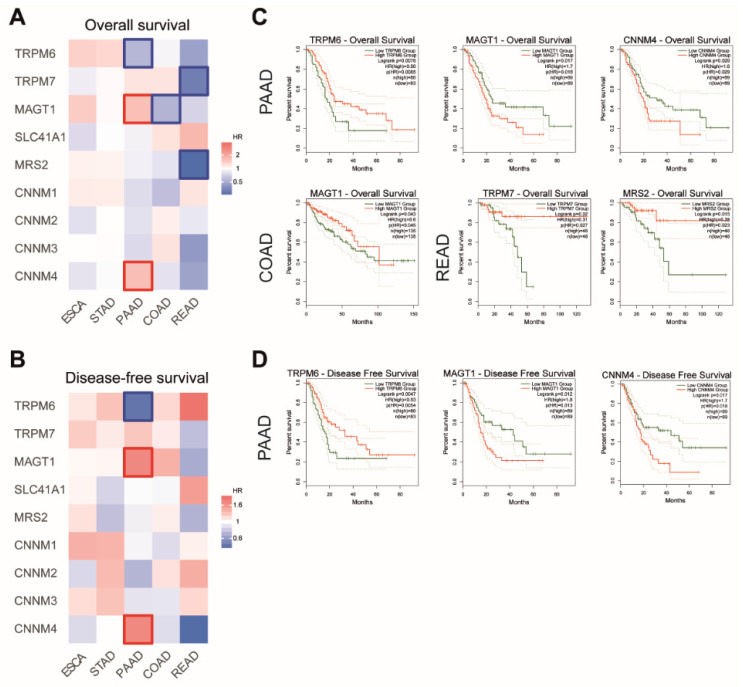
Analysis of patient survival in digestive cancers. Survival heatmaps were generated using GEPIA2 with TCGA data for overall survival (**A**) and disease-free survival (**B**) for Esophageal Cancer (ESCA), Stomach Adenocarcinoma (STAD), Pancreatic Adenocarcinoma (PAAD), Colon Adenocarcinoma (COAD) and Rectum Adenocarcinoma (READ) datasets. Survival is expressed as hazard ratio (HR). Framed squares represent significative statistical values (*p* < *0.05*). Kaplan–Meier curves for overall survival (**C**) and disease-free survival (**D**) were analyzed using GEPIA2. TRPM6, Transient Receptor Potential Cation Channel Subfamily M, Member 6. TRPM7, Transient Receptor Potential Cation Channel Subfamily M, Member 7; MAGT1, Magnesium Transporter 1; SLC41A1, Solute Carrier Family 41, Member 1; MRS2, Mitochondrial RNA Splicing Protein 2; CNNM4, Cyclin and CBS Domain Divalent Metal Cation Transport Mediator 4. Only statistically significant curves (*p* < *0.05*) are presented.

We then searched for a possible association between the Mg transporters’ expression and patient survival. We generated survival heatmaps and Kaplan–Meier curves for overall survival (OS) and disease-free survival (DFS) in TCGA datasets using the GEPIA2 tool (Figure 4).

For PAAD patients, we observed that high expression MAGT1 and CNNM4 mRNA were associated with shorter patient overall survival, while high TRPM6 mRNA expression was correlated with better outcome in those patients. Similar correlations were observed in patients for disease-free survival.

For COAD patients, a high expression of MAGT1 mRNA is associated with a better outcome in patient overall survival.

For READ patients, a longer overall survival is observed when patients have a high expression of TRPM7 and MRS2 mRNA.

Survival analysis of TCGA datasets provides many interesting clues for future research. However, additional analyses of independent cohorts remain mandatory as well as more advanced statistical analysis using R package such as “regnet” in order to increase the robustness of the in silico analysis [87].

#### 4.3.3. Mg^2+^ Transporters and Patient Survival

For each dataset, we studied the co-expression of Mg^2+^ transporters by performing Pearson’s correlation analysis and principal component analysis (PCA) using RStudio (R scripts were previously described in [86]) (Supplementary Tables S1–S4 for the whole analysis).

**Figure 5:**
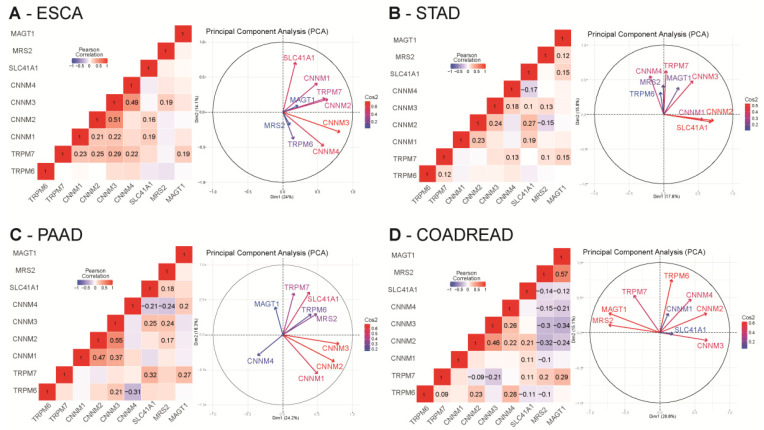
Correlation analysis of relative magnesium transporters mRNA levels in digestive cancers TCGA cohorts. Pearson cor-relation R values were calculated for each magnesium transporter mRNA using RStudio. All queries for TRPM6, TRPM7, MAGT1, SLC41A1, MRS2, CNNM1, CNNM2, CNNM3 and CNNM4 genes were realized in ESCA (**A**), STAD (**B**), PAAD (**C**) and COAD-READ (**D**) datasets from TCGA using the cBioPortal website. mRNA expression values were retrieved as RNA-Seq by Expectation Maximization RSEM (Batch normalized from Illumina HiSeq_RNASeqV2). Only significative correlations values (*p* < 0.05) are presented. Principal component analysis (PCA) of Mg^2+^ transporters mRNA relative expression in TCGA datasets are also represented. Grouped variables are positively correlated whereas opposed variables are negatively correlated. Independency of the variables is formed by a 90° angle formed by two arrows. The quality of the variables on the Principal Component Analysis (PCA) are designated by Cos2 (square cosine, squared coordinates) values.

In esophageal cancer (TCGA-ESCA dataset), we observed a strong positive correlation among CNNM transporters. TRPM7 was also positively correlated with CNNM transporters and MAGT1 (0.19 < R < 0.29). SLC41A1 is positively correlated with CNNM1 (R = 0.19) and CNNM2 (R = 0.16). Relationships between these variables were further confirmed in our PCA plot in which we observed a proximity among CNNM transporters, TRPM7 and MAGT1 (Figure 5A).

In gastric cancer (TCGA-STAD dataset), we observed a mild positive correlation of TRPM7 with TRPM6, MAGT1, CNNM3 and CNNM4 (=0.1< R < 0.22). SLC41A1 is also positively correlated to MAGT1, CNNM1, CNNM2, CNNM3 (R = 0.1–0.27) but negatively correlated to CNNM4 (R = −0.17). MRS2 was positively correlated with TRPM7, CNNM3 and SLC41A1 (R = 0.1–0.13) but negatively correlated to CNNM2 (R = −0.15). CNNM2 is positively correlated with CNNM3 (R = 0.24). Those positive and negative correlations are confirmed in the PCA plot, for example a close proximity of SLC41A1 with CNNM1 and CNNM2 (Figure 5B).

In pancreatic cancer (PAAD dataset), MAGT1 is positively correlated to TRPM7 (R = 0.27) and CNNM4 (R = 0.2). CNNM1, CNNM2 and CNNM3 are also positively correlated with each other (0.17 < R < 0.25). CNNM2, CNNM3, and SLC41A1 are positively correlated to MRS2. SLC41A1 is also positively correlated to CNNM3, and TRPM7. CNNM4 is negatively correlated with SLC41A1 (R = −0.21) and MRS2 (R = −0.24) On the PCA, MAGT1, CNNM4 and TRPM6 are independent (Figure 5C).

In colorectal cancer (COAD-READ dataset), MAGT1 and MRS2 expression are negatively correlated to CNNM2, CNNM3, CNNM4 and SLC41A1 (−0.1 < R < −0.32) but are positively correlated between each other (R = 0.57). TRPM7 is also positively correlated to MAGT1 (R = 0.29), MRS2 (R = 0.2) and SLC41A1 (R = 0.11) but negatively correlated with CNNM2 (R = −0.09) and CNNM3 (R = −0.21). TRPM6 is positively correlated to TRPM7 (R = 0.09), CNNM2 (R = 0.23) and CNNM4 (R = 0.28) but is negatively correlated to SLC41A1 (R = −0.11) and MRS2 (R = −0.1). SLC41A1 is positively correlated to CNNM1 (R = 0.11), CNNM2 (R = 0.21) and TRPM7 (R = 0.11) but negatively correlated to TRPM6 (R = −0.11). On PCA analysis, the strong positive correlation expression of MAGT1 and MRS2 is confirmed and we observed an independency between TRPM6 and CNNM3 and MRS2 (Figure 5D).

## 5. Regulation of Digestive Cancer Cell Fates by Magnesium Transporters

Dysregulation of Mg^2+^ transporters could contribute to cancer hallmarks by regulating malignant cell proliferation, migration or invasion [88].

As mentioned above, TRPM7 overexpression has been proposed as an independent good prognosis biomarker in ESAC. In the TE5 human ESAC cell line, TRPM7 silencing by siRNA enhances cell proliferation as well as migration and invasion [73]. Although the molecular mechanisms involved in TRPM7-mediated ESAC cell proliferation, migration and invasion are far from being fully elucidated, TRPM7 channel expression may prevent the oncogenic properties of ESAC cells.

TRPM7 expression is also detected in GC cell lines but TRPM7 silencing decreases cell viability probably by inducing apoptosis. Interestingly, Mg^2+^ supplementation (10 mM) maintains cell growth when TRPM7 expression is inhibited [89]. Moreover, Mrs2 is upregulated in the multidrug-resistant GC cell line, SGC7901/ADR, increasing adriamycin release and promoting cell growth by p27 downregulation and cyclin D1 upregulation [90].

Similar results are found in PDAC cell lines BxPC-3 and PANC-1 where TRPM7 silencing reduces the cell proliferation by accumulating the cells in G0-G1 phases without affecting the number of apoptotic cells. In high Mg^2+^ culture media, the proliferation of TRPM7-deficient cells is restored [74]. Yee et al. further show that TRPM7 expression is required for preventing BxPC-3 and PANC-1 cells from senescence, and that TRPM7 silencing enhances cytotoxicity induced by gemcitabine treatment [91]. However, it has been also shown that TRPM7 inhibition by using small interfering RNA (siRNA) decreases BxPC-3 cell migration without affecting cell viability [76]. In this study, we show that TRPM7 regulates cation constitutive entry and cytosolic Mg^2+^ levels. Interestingly, external Mg^2+^ supplementation maintains normal cytosolic Mg^2+^ levels in TRPM7-deficient cells, suggesting that TRPM7 silencing may be compensated by others’ Mg^2+^ entry pathways in BxPC-3 cells. Importantly, cell migration is also restored by Mg^2+^ supplementation in TRPM7-deficient BxPC-3, indicating that Mg^2+^ is essential for PDAC cell migration. TRPM7 channels are also required for PDAC cell invasion [75]. We have recently shown that TRPM7 was involved in the secretion of both heat-shock protein 90α (Hsp90α), urokinase plasminogen activator (uPA) and matrix metalloproteinase-2 (MMP-2), leading to enhanced PDAC cell invasion [77]. Moreover, TRPM7 regulates Mg^2+^ constitutive entry and Mg^2+^-dependent cell invasion in the MIA PaCa-2 PDAC cell line. TRPM7 channels are linked to pancreatic carcinogenesis, since they are overexpressed in epithelial pancreatic cells chronically exposed to cadmium pollutant, leading to cytosolic Mg^2+^ accumulation and enhanced cell invasion [92]. Finally, TRPM7 channels are also involved in the interaction between PDAC cells and the tumoral microenvironment because TRPM7 membrane currents and TRPM7-dependent cell migration are both stimulated following treatment with elastin-derived peptides (EDP) that are released by the degradation of the extracellular matrix. Our study suggests that TRPM7 is involved in the response to EDP through its interaction with the ribosomal protein SA (RPSA) [93].

In a colon carcinoma LoVo cell model, cytosolic Mg^2+^ levels are higher in doxorubicin-resistant cells when compared to the doxorubicin-sensitive ones [94]. Nevertheless, resistant cells express less TRPM6 and TRPM7 channels, leading to lower Mg^2+^ influx. TRPM7 silencing induces the acquisition of a more resistant phenotype in sensitive cells, indicating that TRPM7 channel expression is associated with chemoresistance in CRC. Cazzaniga et al. have shown that TRPM7 downregulation was accompanied by the upregulation of MagT1 in doxorubicin-resistant LoVo cells [95]. Moreover, MagT1 silencing strongly inhibits resistant cell proliferation without affecting total intracellular Mg^2+^. Luongo et al. further suggest that TRPM6 and TRPM7 assemblage as heterotetrameric channels regulates Mg^2+^ influx and cell proliferation in the HT-29 CRC cell line [96]. In an azoxymethane-induced colorectal cancer mouse model, the use of the TRPM7 inhibitor waixenicin A induces hypomagnesemia via insufficient Mg^2+^ absorption by the colon. However, neither waixenicin A, nor low Mg^2+^ diet affect the formation of pre-neoplastic lesions in the colon [97]. Furthermore, Huang et al. show that the TRPM7 channel is the main transporter for Mg^2+^ influx in both the HT-29 CRC cell line and in primary mouse colon epithelial cells. Su et al. also show that TRPM7 silencing decreases both HT-29 and SW-480 cell proliferation, migration and invasion [82]. Inhibition of TRPM7 induces the upregulation of E-cadherin and the downregulation of N-cadherin in CRC cells suggesting that this channel may regulate epithelial-to-mesenchymal transition (EMT). Taken together, these results strongly suggest that TRPM7 and Mg^2+^ are not involved in early stages of colon carcinogenesis. On the other hand, TRPM7 is involved in processes occurring at late stages of colon carcinogenesis like EMT, cell migration, invasion and chemoresistance. Recently, Yamazaki et al. have demonstrated that CNNM4 deficiency accelerates epithelial colon cell proliferation in mice [98]. Moreover, primary organoids growth is also enhanced in CNNM4-deficient mice. Intriguingly, capsaicin-stimulated Ca^2+^ influx is also reduced in colonic organoids derived from CNNM4-deficient mice while intracellular [Mg^2+^] is increased. These results suggest a functional interaction between TRPV1 Ca^2+^-channels and CNNM4 in colon. Finally, CNNM4-deficient mice treated with azoxymethane followed by dextran sodium sulfate form more polyps. Importantly, histological analyses of CNNM4-deficient polyps reveal the presence of invading cancer cells. These data clearly show a protective role of CNNM4 expression against colon carcinogenesis.

To resume, Mg^2+^ transporter expression is altered in numerous digestive cancers, leading to dysregulation of cell fates. To date, most of these studies are focused on the TRPM7 channel, but the role of other Mg^2+^ transporters cannot be excluded. However, it still remains unclear if these cancer cell fates may be regulated by intracellular Mg^2+^ homeostasis disturbance, since the role of Mg^2+^ as a second messenger is still being debated.

## 6. Mg^2+^-Regulated Signaling Pathways in Digestive Cancer Cells

In cell-based studies, high Mg^2+^ concentration can cause increased tyrosine-kinases activities. Mg^2+^ is indeed a crucial divalent cation required for the activity of kinases, including non-receptor tyrosine kinases (e.g., Src and Abl Proto-Oncogenes, Janus Kinase (JAK), Focal Adhesion Kinase (FAK), Suppressor Of Cytokine Signaling (SOCS)) and receptor tyrosine kinases (RTKs, such as Growth Factor Receptors (GFR) including VEGFR, EGFR, FGFR, and PDGFR) [99]. Following binding of growth factors to their respective RTKs, cytoplasmic proteins containing Src homology region 2 or phospho-tyrosine-binding domains are recruited to the cell membrane. These recruited proteins either have intrinsic enzymatic activity (such as Src and Phospholipase C (PLC), or serve as docking proteins that are capable to bind additional enzymes [100,101]. Activated RTKs are able to trigger a wide range of downstream signaling pathways, including RAS/RAF/MEK/MAPK, PLC/PKC, PI3K/AKT/mTOR, and JAK/STAT [99].

Although there is scarce information regarding activation of signaling pathways in digestive cancer, recent papers show evidence for the involvement of at least two pathways in these cancers cells: the AKT/mTOR pathway on one hand, and the JAK/STAT pathway on the other hand.

Xie and collaborators were able to show a direct relationship between expression of the SLC41A1 transporter and activation of the AKT/mTOR pathway [78]. SLC41A1 mediates both Mg^2+^ uptake and extrusion [102]. SLC41A1 expression is correlated with clinical outcomes in patients with pancreatic ductal adenocarcinoma, with SLC41A1 being downregulated in tumors. Overexpression of SLC41A1 suppressed orthotopic tumor growth in a mouse model and reduced the cell proliferation, colony formation, and invasiveness of KP3 and Panc-1 cell lines. Overexpression of SLC41A1 promoted Mg^2+^ efflux and suppressed AKT/mTOR activity, which is the upstream regulator of Bax and Bcl-2. An increase in AKT activity and supplementation with Mg^2+^ abolished SLC41A1-induced tumor suppression [78].

At least another Mg^2+^ transporter was found to be associated with the mTOR pathway: CNNM4. This transporter is highly expressed in the colon epithelia, and also strongly expressed in the intestine [58]. In this latter tissue, CNNM4 is localized at the basolateral membrane of epithelial cells and mediates intestinal Mg^2+^ absorption from the tubular lumen across the epithelial sheet, by extruding intracellular Mg^2+^ to the body inside. CNNM4-deficient mice can grow without severe defects but have moderately lowered levels of magnesium in the blood when compared to control wild-type mice due to malabsorption of magnesium [58].

In ApcΔ14/+ mice, which spontaneously form benign polyps in the intestine, deletion of Cnnm4 promoted malignant progression of intestinal polyps to adenocarcinomas. IHC analyses of tissues from patients with colon cancer demonstrated an inverse relationship between CNNM4 expression and colon cancer malignancy, thereby supporting the notion that CNNM4 suppresses the progression of cancer malignancy in humans [84].

CNNM4-dependent Mg^2+^ efflux is apparently able to suppress tumor progression by regulating energy metabolism [84]: Mg^2+^ is able to bind several biomolecules, with ATP being most probably the utmost important. CNNM4 knockdown is able to increase intracellular Mg^2+^ levels, and to significantly increase ATP levels in HEK293 cells. Moreover, CNNM4 knockdown (or Mg^2+^ supplementation) is able to selectively abrogate AMPK hyperphosphorylation (AMPK being phosphorylated and activated under energy-deficient conditions [103]). mTOR is known to be a major downstream target of AMPK signaling [104] and has significant roles in cancer development. Through monitoring of S6K (a well-known substrate of mTOR), Funato and collaborators have clearly identified CNNM4 as a modulator of mTOR signaling [84].

Moreover, it has been shown that Cnnm4 deficiency suppresses Ca^2+^ signaling and promotes cell proliferation in the colon epithelia. These results establish the functional interplay between Mg^2+^ and Ca^2+^ in the colon epithelia, which is crucial for maintaining the dynamic homeostasis of the epithelial tissue [98].

The second signaling pathway influenced by magnesium levels is the STAT pathway. This influence occurs through Magnesium-dependent Phosphatase (MDP)-1. This enzyme, belonging to the haloacid dehalogenase family, has a protein–tyrosine phosphatase function and is potentially involved in glycation repair (Fortpied et al., 2006).

Forced expression of MDP1 in the gastric cancer cell line BGC-823 inhibited cell proliferation, whereas the knockdown of MDP1 protein promoted cell growth. Overexpression of MDP1 in BGC-823 cells also enhanced cell senescence and apoptosis. Signal transducer and activator of transcription 3 (*Stat3*), as well as the c-Jun *N*-terminal kinase (JNK) were found to mediate the biological function of MDP1 [105].

TRPM7 has also been linked to Stat3: disrupted expression of Trpm7 in mice causes downregulated expression of *Stat3* mRNA [106].

**Figure 6:**
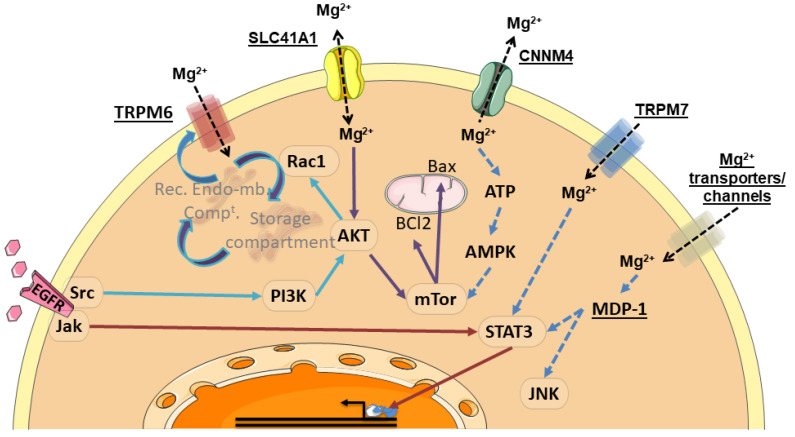
Summary of signaling pathways associated with Mg^2+^ transporters. Mg^2+^ transporter, channels and Mg^2+^-dependent proteins are underlined. These proteins activate kinases, signaling proteins and/or transcription factors (framed in ovals). As a result, some processes, such as protein redistribution to the plasma membrane, control of effector proteins and transcriptional regulation can occur. Rec. Endo-mb. Comp^t^.: recycling endomembrane compartment. Mg^2+^, Magnesium; EGFR, Epidermal Growth Factor Receptor; TRPM6, Transient Receptor Potential Cation Channel Subfamily M, Member 6; TRPM7, Transient Receptor Potential Cation Channel Subfamily M, Member 7; SLC41A1, Solute Carrier Family 41, Member 1; CNNM4, Cyclin and CBS Domain Divalent Metal Cation Transport Mediator 4; Jak, Janus Kinase; Src, Src Proto-Oncogene; PI3K, Phosphatidylinositol-4,5-Biphosphate 3-Kinase; AKT, AKT Serine/Threonine Kinase; Rac1, Rac-Family Small GTPase 1; mTor, Mechanistic Target of Rapamycin Kinase; BCl2, BCL2 Apoptosis Regulator; Bax, BCL2 Associated X Apoptosis Regulator; ATP, Adenosine Tri-Phosphate; AMPK, AMP-Activated Protein Kinase, STAT3, Signal Transducer And Activator Of Transcription 3; MDP-1, Magnesium-Dependent Phosphatase-1; JNK, c-Jun *N*-terminal Kinase.

Finally, it has to be mentioned that anti-RTK EGFR antibodies are able to dramatically reduce serum magnesium concentration [107,108]. Although EGFR tyrosine kinase inhibitors can also potentially induce hypomagnesaemia, typical concentrations used in the clinic seem to be insufficient to induce this side-effect [108]. Inhibition of the EGFR induces a mutated-like TRPM6 syndrome [108], while stimulation of the EGFR increase current through TRPM6 (but not TRPM7) [109]. The α-kinase domain of TRPM6 is not involved in the EGF receptor-mediated increase in channel activity: the activation relies on both Src and the downstream effector Rac1, the latter being able to increase TRPM6 mobility and increase cell surface abundance by redistributing endomembrane TRPM6 to the plasma membrane [109] (Figure 6).

Other transporters have been demonstrated to activate magnesium-dependent signaling pathways, such as MAGT1. Indeed, Li and collaborators have identified mutations in this magnesium transporter gene, in a novel X-linked human immunodeficiency characterized by CD4 lymphopenia, severe chronic viral infections, and defective T lymphocyte activation [50]. MAGT1 deficiency was shown to abrogate Mg^2+^ influx, leading to defective activation of phospholipase Cγ and consequently impaired responses to antigen receptor engagement in patients harboring this XMEN (X-link immunodeficiency with Magnesium defect and EBV infection and Neoplasia) disease. However, it must be noted (i) that Mg^2+^ supplementation has not proven successful [110], and (ii) that XMEN disease has been recently shown to be a congenital disorder of glycosylation that affects a restricted subset of glycoproteins. MAGT1 is actually a non-catalytic subunit required for N-glycosylation of key immune cells receptors [111]. The mechanism by which MAGT1 is involved in the magnesium homeostasis and how magnesium affects glycosylation requires further investigation [110].

## 7. Conclusions and Perspectives

**Figure 7:**
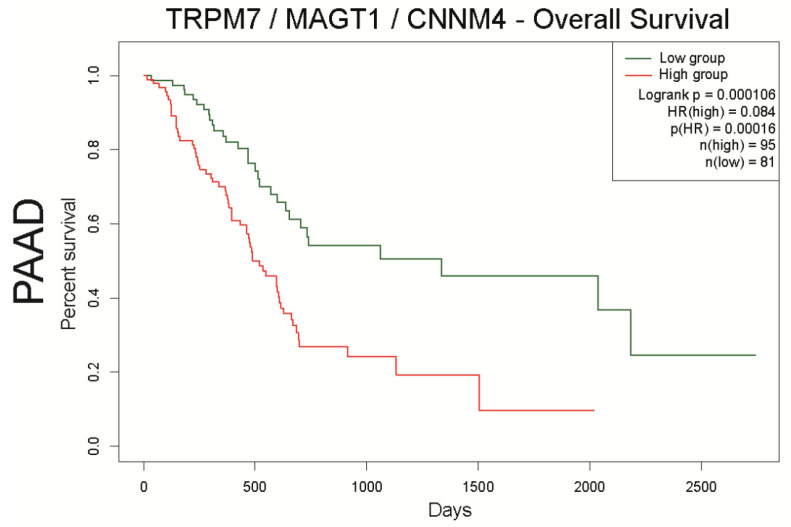
Analysis of overall survival of the TRPM7/MAGT1/CNNM4 signature in pancreatic cancer datasets using SurvExpress. PAAD patients were stratified using a gene signature combining TRPM7, MAGT1, and CNNM4. Kaplan–Meier curves were analyzed using the optimized SurvExpress Maximize algorithm. The number of analyzed patients across time (days) is indicated below the horizontal axis for both conditions, as previously described [86].

The aim of this work was to make an overview of Mg^2+^ transporter expression and their role in esophageal adenocarcinoma (ESAC), gastric cancer (GC), pancreatic ductal adenocarcinoma (PDAC), and colorectal cancer (CRC). These digestive cancers are the most common and the deadliest malignancies worldwide. Numerous epidemiologic studies strongly suggest that digestive cancer incidence and mortality may be dependent on lifestyle, and particularly diet. Mg^2+^ content is continuously decreased in alimentation of industrialized countries, leading to nutritional deficiency in Mg^2+^ for a large part of the population, estimated to ~60% in the USA [112]. While Mg^2+^ is the second most abundant cation in the cell, its role in physiology and pathology is less extensively studied than others such as Ca^2+^, Na^+^ or K^+^. Cellular Mg^2+^ homeostasis is regulated by membrane transporters. Among these transporters, TRPM7 has been clearly identified as the main Mg^2+^ gatekeeper for cell intake in both non-cancer and cancer cells. On the other hand, the functional characterization of other candidates such as MAGT1 or CNNM4 as Mg^2+^ transporter is still under debate. The reviewed literature, as well as the Human Protein Atlas analyses indicate that Mg^2+^ transporter expression is altered in most digestive cancers. The discrepancies between the data could be explained by the low number of cohorts and/or by the methodology used. In particular, antibodies targeting ion channels and transporters often display a poor specificity, inducing potential cross-reactivity with other molecules [113]. Analyses of Mg^2+^ transporter expression in cells and tissues by immunochemistry should be systematically completed by other methods of detection such as functional (e.g., electrophysiology and Mg^2+^-imaging) and transcriptomic analysis. In this work, we analyzed Mg^2+^ transporters expression in digestive cancers, correlation and their impact on patient overall and disease-free survival using Genotype Tissue Expression (GTEx) and The Cancer Genome Atlas (TCGA), as previously described [86]. Interestingly, our data reveal that the MAGT1 is overexpressed in all digestive cancers. Moreover, MAGT1 expression is associated with a poor survival in PDAC patients and with a better survival in CRC patients. Cazzaniga et al. show that MAGT1 is overexpressed in a CRC cell line resistant to doxorubicin compared to the sensitive ones, and it regulates cell proliferation [95]. To our knowledge, the role of MAGT1 has not been yet studied in other digestive cancer cell models and further investigations are needed to better understand the mechanisms involving this protein in digestive cancers. It has been shown that MAGT1 expression can restore TRPM7 deficiency, intracellular Mg^2+^ homeostasis and cell viability in some cellular models suggesting a possible transcriptomic regulation [95,114]. Interestingly, our data show a positive correlation between TRPM7/MAGT1/CNNM4 expression in both ESAC and PDAC, and also between TRPM7 and MAGT1 expression in CRC. This suggests a possible interaction between these three proteins in ESAC and PDAC cancer cells. In our opinion, such complex may be of great interest for the research of new biomarkers, especially for PDAC as high expression of TRPM7/MAGT1/CNNM4 clearly discriminates a unique profile of patients with the poorest survival (Figure 7). We found that TRPM7/MAGT1/CNNM4 high expression is also associated with a poor survival in the other digestive cancers (Supplementary Figure S2). However, these results are only preliminary and should be confirmed by additional analyses on independent datasets, as well as IHC on FFPE. TRPM7 possess a functional kinase domain able to phosphorylate serine or threonine residues, while MAGT1 is implicated in protein glycosylation. Additionally, it has been suggested that CNNM4 expression could regulate TRP channel expression such as TRPV1 [98]. Therefore, it is possible that transcriptomic regulations and/or post-translational modifications can occur between TRPM7, MAGT1 and CNNM4 in digestive cancer cells.

To conclude, this review highlights Mg^2+^ transporters and their associated signaling pathways as promising biomarkers in digestive cancers. The transcriptomic analysis of datasets reveals a Mg^2+^ transporter signature involving TRPM7/MAGT1/CNNM4 in ESAC and PDAC. In PDAC, the correlation between TRPM7, MAGT1 and CNNM4 expression is associated with a low survival. Further studies are needed to better understand the physiological significance of this complex in cancer cell models.

## Figures and Tables

**Table 1:** Expression of Mg^2+^ Transporters in Digestive Cancer Tissues.

Cancer	Transporter	Expression in Cancerous Tissues (Compared to Normal Tissues)	Technique	Reference
ESAC	TRPM7	Upregulated	IHC	[73]
PDAC	TRPM7	Upregulated	IHC	[74,75,76,77]
	SLC41A1	Downregulated	In silico + Protein Atlas qRT-PCR Western-Blot	[78]
CRC	TRPM6	Downregulated (RNA) Upregulated (Protein)	In silico qRT-PCR IHC	[79,80,81]
	TRPM7	Upregulated	In silico qRT-PCR IF IHC	[81,82]
	MAGT1	Upregulated	In silico qRT-PCR	[83]
	CNNM4	Downregulated	IHC	[84]

ESAC, Esophageal Cancer; PDAC, Pancreatic Ductal Adenocarcinoma; CRC, Colorectal Cancer; MAGT1, Magnesium Transporter 1; SLC41A1, Solute Carrier Family 41 Member 1; TRPM7, Transient Receptor Potential Cation Channel Subfamily M Member 7; TRPM6, Transient Receptor Potential Cation Channel Subfamily M Member 6; CNNM4, Cyclin and CBS Domain Divalent Metal Cation Transport Mediator4; IHC, Immunohistochemistry; qRT-PCR, quantitative RT-PCR; IF, Immunofluorescence.

**Table 2:** Expression of Magnesium transporters using immunohistochemistry (IHC) in some digestive cancers based on The Human Protein Atlas data.

Transporter	GC	PDAC	CRC
TRPM7	33%	0%	0%
MAGT1	44.4%	22.2%	83.3%
SLC41A1	91.7%	100%	100%
MRS2	75%	91.7%	75%
CNNM:			
CNNM1 CNNM3 CNNM4	12.5% 72.7% 50%	36.4% 100% 58.3%	16.7% 100% 83.3%

Percentages of tumoral tissue samples with strong and moderate staining using IHC were calculated from the protein atlas data [85]. Only data for Pancreatic Ductal Adenocarcinoma (PDAC), Colorectal Cancer (CRC) and Gastric Cancer (GC) were available and for each transporters a number of patients ranging between 8 and 12 was tested. CNNM1/2/3/4, Cyclin and CBS Domain Divalent Metal Cation Transport Mediator1/2/3/4; MAGT1, Magnesium Transporter 1; SLC41A1, Solute Carrier Family 41 Member 1; TRPM7, Transient Receptor Potential Cation Channel Subfamily M Member 7; TRPM6, Transient Receptor Potential Cation Channel Subfamily M Member 6; CNNM1, Cyclin And CBS Domain Divalent Metal Cation Transport Mediator1; MRS2, Mitochondrial RNA Splicing Protein 2. No data were found for TRPM6 and CNNM2 expression.

## Data Availability

All data are available and are based upon public data extracted from TCGA Research Network (http://cancergenome.nih.gov/), Genome Tissue Expression (GTEX) project (http://www.GTEXportal.org/), and Gene Expression Omnibus (GEO) database (http://www.ncbi.nml.nih.gov/geo/) using GEPIA2 (http://gepia2.cancer-pku.cn/) and SurvExpress (SurvExpress—Web resource for Biomarker comparison and validation of Survival gene eXpression data in cancer (itesm.mx)).

## Supplementary Materials

The following are available online at https://www.mdpi.com/2072-6643/13/1/210/s1, Figure S1: Relative mRNA expression of magnesium transporters in digestive cancers and normal tissues. Figure S2: Analysis of overall survival of the TRPM7/MAGT1/CNNM4 signature in digestive cancer datasets using SurExpress (ESCA for esophageal carcinoma (A); STAD for stomach adenocarcinoma (B); PAAD for pancreatic adenocarcinoma (C); COADREAD for colorectal adenocarcinoma (D). Table S1. Pearson correlation of magnesium transporters combination in esophageal cancer (ESCA) dataset. Table S2. Pearson correlation of magnesium transporters combination in stomach cancer (STAD) dataset. Table S3. Pearson correlation of magnesium transporters combination in pancreatic cancer (PAAD) dataset. Table S4. Pearson correlation of magnesium transporters combination in colorectal cancer (COADREAD) dataset.
