# Supplementary material for: Mg2+ Transporters in Digestive Cancers
Source: Nutrients. 2021 Jan 13;13(1):210. doi: 10.3390/nu13010210 (PMC7828344; doi:10.3390/nu13010210)
Supplement: Supplementary file 1 [file nutrients-13-00210-s001.pdf]

nutrients

MDPI

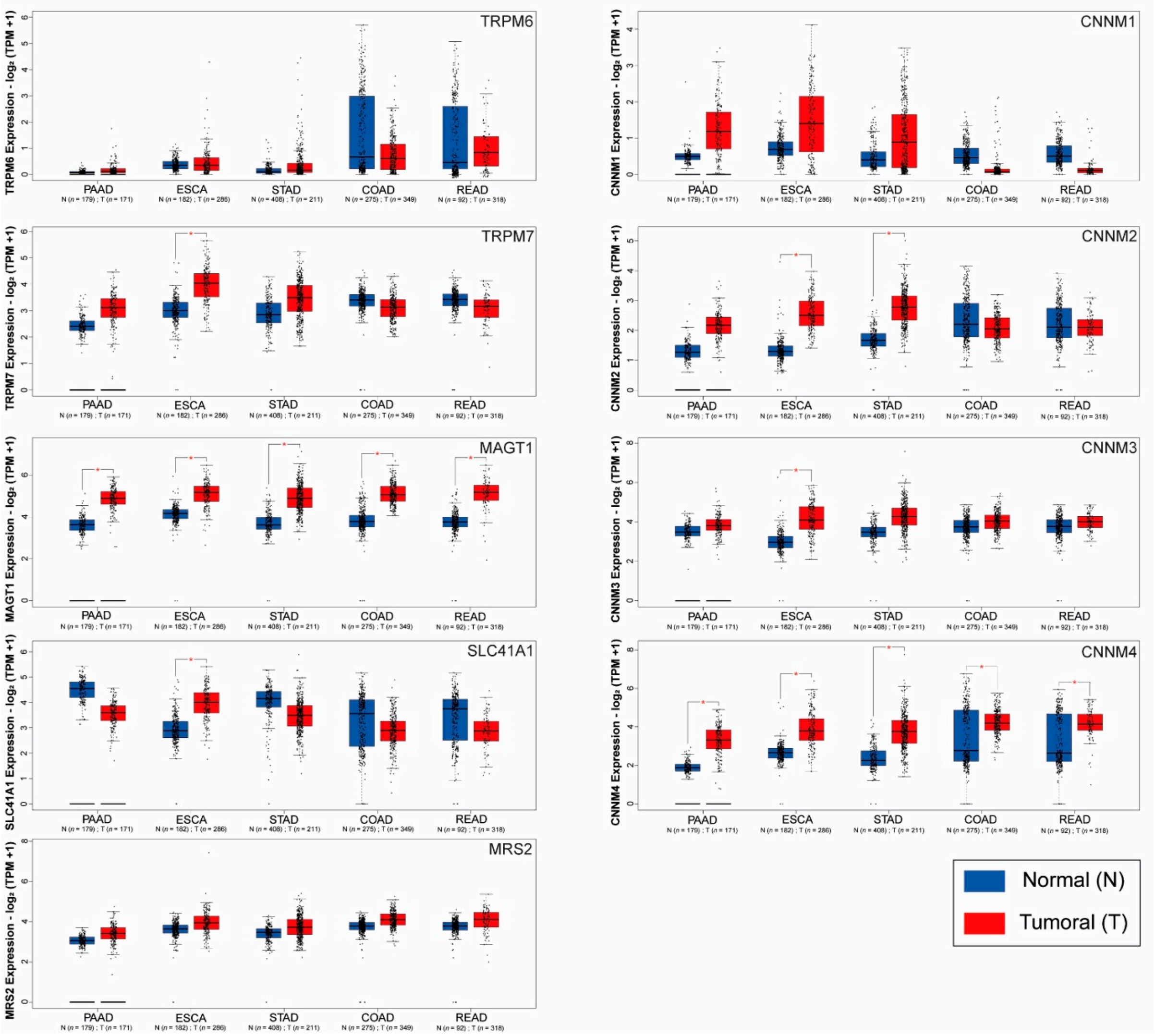

Supplementary Figure S1. Relative mRNA expression of magnesium transporters in digestive cancers and normal tissues. Whiskers boxplots for Mg^{2+} transporters mRNA (TRPM6, TRPM7, MAGT1, SLC41A1, MRS2, CNNM1, CNNM2, CNNM3, CNNM4) were generated using GEPIA2 (http://gepia2.cancer-pku.cn/) in The Cancer Genome Atlas (TCGA) and Genotype-Tissue Expression (GTEx) samples. TCGA datasets ESCA, STAD, PAAD, COAD and READ. Relative mRNA levels are expressed as log2 Transcripts Per Million bases (TPM) (* $p < 0.01$). ESCA, Esophageal Cancer; STAD, Stomach Adenocarcinoma; PAAD, Pancreatic Adenocarcinoma; COAD, Colon Adenocarcinoma; READ, Rectum Adenocarcinoma; TRPM6, Transient Receptor Potential Cation Channel Subfamily M, Member 6; TRPM7, Transient Receptor Potential Cation Channel Subfamily M, Member 7; MAGT1, Magnesium Transporter 1; SLC41A1, Solute Carrier Family 41, Member 1; MRS2, Mitochondrial RNA Splicing Protein 2; CNNM1, Cyclin And CBS Domain Divalent Metal Cation Transport Mediator 1; CNNM2, Cyclin And CBS Domain Divalent Metal Cation Transport Mediator 2; CNNM3, Cyclin and CBS Domain Divalent Metal Cation Transport Mediator 3; CNNM4, Cyclin And CBS Domain Divalent Metal Cation Transport Mediator 4; *n* = number of samples; N, normal; T, tumoral.

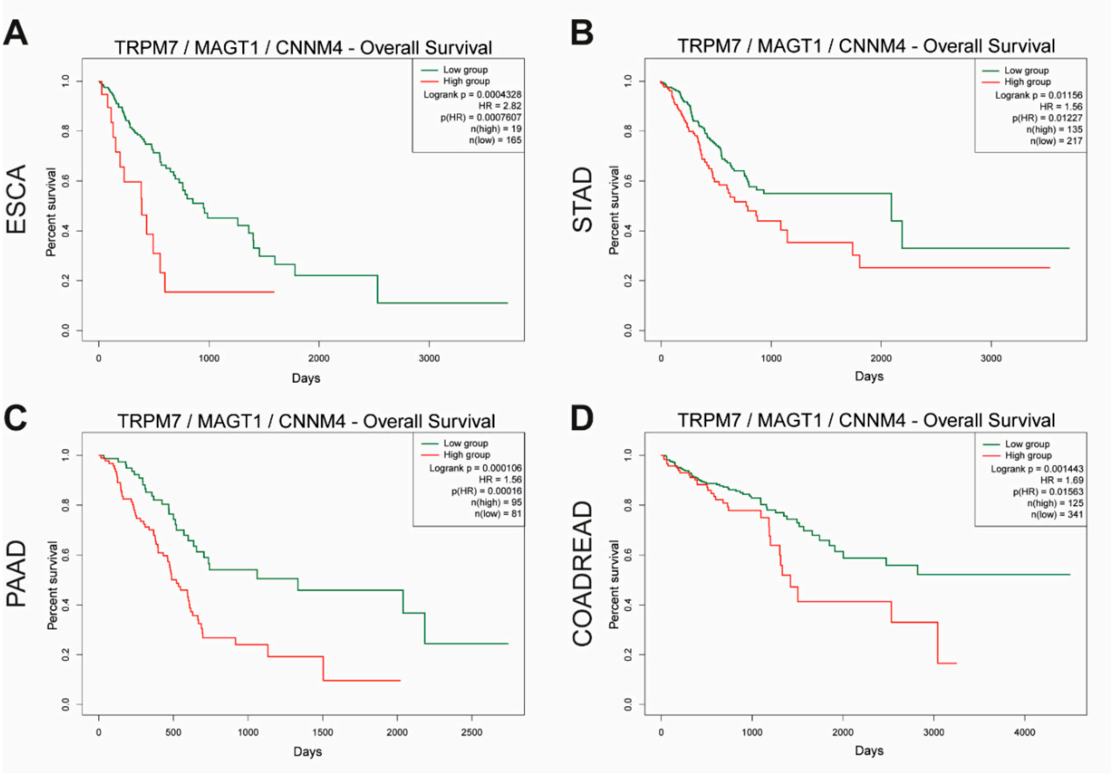

Supplementary Figure S2. Analysis of overall survival of the TRPM7/MAGT1/CNNM4 signature in digestive cancer datasets using SurvExpress (ESCA for Esophageal Cancer (**A**); STAD for Stomach Adenocarcinoma (**B**); PAAD for pancreatic adenocarcinoma (**C**); COADREAD for Colorectal Adenocarcinoma (**D**). TRPM7, Transient Receptor Potential Cation Channel Subfamily M, Member 7; MAGT1, Magnesium Transporter 1; CNNM4, Cyclin And CBS Domain Divalent Metal Cation Transport Mediator 4; HR, Hazard Ratio.

Supplementary Table S1. Pearson correlation of magnesium transporters combination in esophageal cancer (ESCA) dataset.

ESCA			
**Gene 1**	**Gene 2**	**Pearson Correlation (R)**	***p*-Value**
CNNM2	CNNM3	0.51	4.3587E-13
CNNM3	CNNM4	0.49	4.2242E-12
TRPM7	CNNM3	0.29	7.3238E-05
TRPM7	CNNM2	0.25	0.00082929
TRPM7	CNNM1	0.23	0.00216094
TRPM7	CNNM4	0.22	0.00297761
CNNM1	CNNM3	0.22	0.00337917
CNNM1	CNNM2	0.21	0.0052659
CNNM1	SLC41A1	0.19	0.0105924
CNNM3	MRS2	0.19	0.01129643
TRPM7	MAGT1	0.19	0.01150486
CNNM2	SLC41A1	0.16	0.03428567
CNNM1	CNNM4	0.13	0.09030724
TRPM7	SLC41A1	0.12	0.10168617
TRPM6	CNNM4	0.12	0.12378106
TRPM6	CNNM3	0.11	0.14785956
CNNM2	CNNM4	0.09	0.21010962
CNNM3	MAGT1	0.09	0.2253436
CNNM4	MRS2	0.08	0.2696619
CNNM1	MAGT1	0.08	0.27587911
SLC41A1	MRS2	0.05	0.47652983
TRPM6	CNNM2	0.05	0.51875554
CNNM4	MAGT1	0.05	0.5445937
MRS2	MAGT1	0.04	0.58635219
TRPM6	TRPM7	0.02	0.75575179
SLC41A1	MAGT1	0.01	0.86656615
TRPM6	CNNM1	0.01	0.90546796
CNNM2	MAGT1	−0.01	0.9294394
TRPM6	MAGT1	−0.01	0.93193885
CNNM3	SLC41A1	−0.02	0.80870089
TRPM7	MRS2	−0.03	0.68302553
CNNM1	MRS2	−0.06	0.38681974
CNNM2	MRS2	−0.06	0.45803698
CNNM4	SLC41A1	−0.07	0.3506591
TRPM6	SLC41A1	−0.08	0.29515527
TRPM6	MRS2	−0.11	0.12728537

Pearson correlation of magnesium transporters combination in Esophageal Cancer (ESCA) dataset. Significative Pearson correlation values are presented in red. TRPM6, Transient Receptor Potential Cation Channel Subfamily M, Member 6; TRPM7, Transient Receptor Potential Cation Channel Subfamily M, Member 7; MAGT1, Magnesium Transporter 1; SLC41A1, Solute Carrier Family 41, Member 1; MRS2, Mitochondrial RNA Splicing Protein 2; CNNM1, Cyclin And CBS Domain Divalent Metal Cation Transport Mediator 1; CNNM2, Cyclin And CBS Domain Divalent Metal Cation Transport Mediator 2; CNNM3, Cyclin and CBS Domain Divalent Metal Cation Transport Mediator 3; CNNM4, Cyclin And CBS Domain Divalent Metal Cation Transport Mediator 4.

Supplementary Table S2. Pearson correlation of magnesium transporters combination in stomach cancer (STAD) dataset.

		STAD	
**Gene 1**	**Gene 2**	**Pearson Correlation (R)**	***p*-Value**
CNNM2	SLC41A1	0.27	2.8926E-08
CNNM2	CNNM3	0.24	5.9936E-07
CNNM1	CNNM2	0.23	4.605E-06
CNNM1	SLC41A1	0.19	0.00014494
CNNM3	CNNM4	0.18	0.0002445
TRPM7	MAGT1	0.15	0.00185495
SLC41A1	MAGT1	0.15	0.00286807
TRPM7	CNNM4	0.13	0.00820752
CNNM3	MRS2	0.13	0.01152511
MRS2	MAGT1	0.12	0.01389851
TRPM6	TRPM7	0.12	0.01674765
TRPM7	MRS2	0.1	0.0358132
CNNM3	SLC41A1	0.1	0.04881246
TRPM7	CNNM3	0.1	0.05041349
TRPM6	CNNM4	0.07	0.1637985
CNNM1	CNNM3	0.07	0.1729782
CNNM1	MAGT1	0.05	0.30501749
TRPM6	CNNM3	0.04	0.40372046
TRPM6	CNNM2	0.04	0.4263219
CNNM4	MAGT1	0.03	0.50062739
CNNM1	MRS2	0.03	0.56009289
CNNM3	MAGT1	0.03	0.57354939
SLC41A1	MRS2	0.01	0.87851642
CNNM4	MRS2	0	0.94083231
TRPM7	SLC41A1	0	0.95060405
TRPM6	MAGT1	0	0.96534282
TRPM6	MRS2	−0.01	0.78816561
CNNM2	MAGT1	−0.01	0.79656385
TRPM7	CNNM2	−0.01	0.91628163
TRPM7	CNNM1	−0.03	0.59819559
TRPM6	CNNM1	−0.04	0.36808418
CNNM1	CNNM4	−0.04	0.37498868
TRPM6	SLC41A1	−0.06	0.22686941
CNNM2	CNNM4	−0.09	0.07758721
CNNM2	MRS2	−0.15	0.00191779
CNNM4	SLC41A1	−0.17	0.00060241

Pearson correlation of magnesium transporters combination in Stomach Adenocarcinoma (STAD) dataset. Significative Pearson correlation values are presented in red. TRPM6, Transient Receptor Potential Cation Channel Subfamily M, Member 6; TRPM7, Transient Receptor Potential Cation Channel Subfamily M, Member 7; MAGT1, Magnesium Transporter 1; SLC41A1, Solute Carrier Family 41, Member 1; MRS2, Mitochondrial RNA Splicing Protein 2; CNNM1, Cyclin And CBS Domain Divalent Metal Cation Transport Mediator 1; CNNM2, Cyclin And CBS Domain Divalent Metal Cation Transport Mediator 2; CNNM3, Cyclin and CBS Domain Divalent Metal Cation Transport Mediator 3; CNNM4, Cyclin And CBS Domain Divalent Metal Cation Transport Mediator 4.

Supplementary Table S3. Pearson correlation of magnesium transporters combination in pancreatic cancer (PAAD) dataset.

PAAD			
**Gene 1**	**Gene 2**	**Pearson Correlation (R)**	***p*-Value**
CNNM2	CNNM3	0.55	1.8208E-14
CNNM1	CNNM2	0.47	8.5755E-11
CNNM1	CNNM3	0.37	1.0627E-06
TRPM7	SLC41A1	0.32	2.8961E-05
TRPM7	MAGT1	0.27	0.00046809
CNNM3	SLC41A1	0.25	0.00090308
CNNM3	MRS2	0.24	0.00207141
TRPM6	CNNM3	0.21	0.0053317
CNNM4	MAGT1	0.2	0.00922737
SLC41A1	MRS2	0.18	0.01725179
CNNM2	MRS2	0.17	0.02636835
TRPM7	MRS2	0.15	0.05283531
TRPM6	SLC41A1	0.12	0.12
SLC41A1	MAGT1	0.12	0.12507537
CNNM1	CNNM4	0.12	0.12802338
TRPM6	CNNM2	0.12	0.13604687
TRPM6	MRS2	0.11	0.14762385
TRPM6	TRPM7	0.1	0.19879538
TRPM7	CNNM3	0.09	0.24771628
CNNM2	SLC41A1	0.08	0.31322368
CNNM1	MRS2	0.07	0.36277495
TRPM7	CNNM4	0.06	0.45420615
MRS2	MAGT1	0.06	0.4752441
TRPM6	MAGT1	−0.01	0.88404109
TRPM6	CNNM1	−0.02	0.80562682
TRPM7	CNNM1	−0.02	0.83076326
CNNM1	MAGT1	−0.06	0.44308262
CNNM2	MAGT1	−0.07	0.38837676
TRPM7	CNNM2	−0.08	0.29059499
CNNM3	CNNM4	−0.09	0.22687325
CNNM2	CNNM4	−0.1	0.19999921
CNNM3	MAGT1	−0.11	0.15748134
CNNM1	SLC41A1	−0.13	0.10609665
CNNM4	SLC41A1	−0.21	0.00656179
CNNM4	MRS2	−0.24	0.00183471
TRPM6	CNNM4	−0.31	3.7043E−05

Pearson correlation of magnesium transporters combination in Pancreatic Adenocarcinoma (PAAD) dataset. Significative Pearson correlation values are presented in red. TRPM6, Transient Receptor Potential Cation Channel Subfamily M, Member 6; TRPM7, Transient Receptor Potential Cation Channel Subfamily M, Member 7; MAGT1, Magnesium Transporter 1; SLC41A1, Solute Carrier Family 41, Member 1; MRS2, Mitochondrial RNA Splicing Protein 2; CNNM1, Cyclin And CBS Domain Divalent Metal Cation Transport Mediator 1; CNNM2, Cyclin And CBS Domain Divalent Metal Cation Transport Mediator 2; CNNM3, Cyclin and CBS Domain Divalent Metal Cation Transport Mediator 3; CNNM4, Cyclin And CBS Domain Divalent Metal Cation Transport Mediator 4.

Supplementary Table S4. Pearson correlation of magnesium transporters combination in colorectal cancer (COADREAD) dataset.

		COADREAD	
**Gene 1**	**Gene 2**	**Pearson Correlation (R)**	***p*–Value**
MRS2	MAGT1	0.57	<1.77636E−15
CNNM2	CNNM3	0.46	<1.77636E−15
TRPM7	MAGT1	0.29	8.29425E−12
TRPM6	CNNM4	0.28	6.45928E−11
CNNM3	CNNM4	0.26	1.94922E−09
TRPM6	CNNM2	0.23	7.27686E−08
CNNM2	CNNM4	0.22	3.47689E−07
CNNM2	SLC41A1	0.21	1.71773E−06
TRPM7	MRS2	0.2	2.27227E−06
TRPM7	SLC41A1	0.11	0.010540664
CNNM1	SLC41A1	0.11	0.012024217
TRPM6	TRPM7	0.09	0.033597761
TRPM7	CNNM1	0.09	0.051725062
CNNM1	CNNM2	0.08	0.057659131
TRPM6	CNNM1	0.06	0.16070676
TRPM6	MAGT1	0.02	0.638185705
CNNM1	CNNM3	0.02	0.657194608
CNNM1	CNNM4	0.02	0.719541484
CNNM3	SLC41A1	0.01	0.857871166
TRPM7	CNNM4	−0.01	0.848984773
CNNM1	MAGT1	−0.03	0.457967958
CNNM4	SLC41A1	−0.04	0.384206948
TRPM6	CNNM3	−0.07	0.103558442
TRPM7	CNNM2	−0.09	0.030191538
CNNM1	MRS2	−0.1	0.023737824
TRPM6	MRS2	−0.1	0.027251471
TRPM6	SLC41A1	−0.11	0.01037094
SLC41A1	MAGT1	−0.12	0.006628715
SLC41A1	MRS2	−0.14	0.001072695
CNNM4	MRS2	−0.15	0.000428972
TRPM7	CNNM3	−0.21	8.04047E−07
CNNM4	MAGT1	−0.21	1.77909E−06
CNNM2	MAGT1	−0.24	1.76794E−08
CNNM3	MRS2	−0.3	1.20171E−12
CNNM2	MRS2	−0.32	3.06422E−14
CNNM3	MAGT1	−0.34	1.77636E−15

Pearson correlation of magnesium transporters combination in Colorectal Adenocarcinoma (COADREAD) dataset. Significative Pearson correlation values are presented in red. TRPM6, Transient Receptor Potential Cation Channel Subfamily M, Member 6; TRPM7, Transient Receptor Potential Cation Channel Subfamily M, Member 7; MAGT1, Magnesium Transporter 1; SLC41A1, Solute Carrier Family 41, Member 1; MRS2, Mitochondrial RNA Splicing Protein 2; CNNM1, Cyclin And CBS Domain Divalent Metal Cation Transport Mediator 1; CNNM2, Cyclin And CBS Domain Divalent Metal Cation Transport Mediator 2; CNNM3, Cyclin and CBS Domain Divalent Metal Cation Transport Mediator 3; CNNM4, Cyclin And CBS Domain Divalent Metal Cation Transport Mediator 4.
